# Continuity of Firearm Injury Documentation From Acute Care to Ambulatory Care Among Medicaid Enrollees in Oregon

**DOI:** 10.1016/j.amepre.2025.108254

**Published:** 2025-12-26

**Authors:** Natalie Cartwright, Alan Cook, Frances M. Biel, Kerime Toksu, David Hosmer, Turner Osler, Megan Hoopes, Nicole Cook

**Affiliations:** 1 Department of Mathematics, Norwich University, Northfield, Vermont; 2 Center for Clinical Research, The University of Texas at Tyler, Tyler, Texas; 3 OCHIN, Inc., Portland, Oregon; 4 Independent consultant, Cape Canaveral, Florida; 5 Department of Mathematics and Statistics, University of Massachusetts at Amherst, Amherst, Massachusetts; 6 The Robert Larner College of Medicine, University of Vermont, Burlington, Vermont

## Abstract

**Introduction:**

Gunshot wounds are typically treated in acute care settings, but longer-term sequelae are likely treated by ambulatory care providers. To understand whether gunshot wounds treated in acute care settings are later documented in ambulatory care electronic health records, acute care claims and ambulatory care electronic health records data were linked. The primary objective was to determine the percentage of patients with a gunshot wound diagnosis in their ambulatory care electronic health records from all patients with both an acute care gunshot wound diagnosis and an observed follow-up ambulatory care visit. The second objective was to identify demographic and clinical factors associated with a patient having a gunshot wound diagnosis in their ambulatory care electronic health records.

**Methods:**

This observational study linked Oregon Medicaid claims for acute gunshot wound with ambulatory care electronic health records in OCHIN’s ADVANCE Data Warehouse from 2012 to 2022 for case identification. Patients with and without an International Classification of Diseases code for gunshot wound in their ambulatory care electronic health records were characterized using descriptive statistics. Multivariable logistic regression conducted between 2024 and 2025 modeled predictors of gunshot wound diagnosis in follow-up ambulatory care electronic health records controlling for patient characteristics.

**Results:**

Only 34.3% of patients with a gunshot wound event treated in an acute setting and with an observed ambulatory care visit had a gunshot wound diagnosis in their ambulatory care electronic health records. Time between acute gunshot wound and follow-up ambulatory care and the severity of the acute gunshot wound injury were associated with having a gunshot wound ambulatory care electronic health records diagnosis. Patients identified as Black, non-Hispanic or Hispanic had higher odds of having a gunshot wound ambulatory care electronic health records diagnosis than those identified as White, non-Hispanic.

**Conclusions:**

Most people who had a gunshot wound treated in an acute care setting with a later ambulatory care visit did not have a gunshot wound diagnosis in their ambulatory care electronic health records, even though a gunshot wound event can be relevant history for ambulatory care providers and patient care. Strategies to document prior gunshot wound in ambulatory electronic health records should be developed to support whole-person care in ambulatory settings.

## INTRODUCTION

Firearm violence is a public health crisis in the U.S., of which gunshot wounds (GSWs) are the principal component, estimated to be more than double the amount of firearm fatalities.^1^ Precise estimates are not possible because there is no database or national system that accurately tracks nonfatal GSWs.^2^ It is estimated that 90% of patients with nonfatal GSWs are first seen in acute care settings, and thus, most GSW data come from emergency department (ED) or inpatient (IP) hospital encounters.^3^ However, these data on acute GSWs can only provide a count of nonfatal GSW; it alone cannot be used to study long-term health sequelae associated with nonfatal GSWs; for this, ambulatory care data are needed.

Ambulatory/outpatient data (e.g., ambulatory care electronic health records [acEHRs] from primary or behavioral health care) are essential in the study of health outcomes, healthcare utilization, and healthcare costs associated with GSWs. However, there is limited understanding of how ambulatory care settings provide care to and subsequently document their care of patients with GSWs. After acute GSW, patients may receive follow-up care in ambulatory care settings for bodily trauma (e.g., removal of sutures), treatment for GSW that was not seen in an ED or IP setting (e.g., grazing of bullet), or other health impacts after GSW (e.g., pain care, post-traumatic stress disorder, substance use or abuse, sleeping disorders).^4,5^ Documentation of care may include International Classification of Diseases (ICD) codes for GSWs, although ICD codes that are used in acute care may not be applicable to many ambulatory encounters.^6^ For example, if the care received is for a behavioral health condition associated with GSW trauma, then a diagnosis code of GSW may not be assigned, especially if the code is not relevant to billing. Likewise, it is unknown whether a GSW code is entered into a patient’s medical history (i.e., problem list) when a patient mentions having a GSW or is seen for an associated health issue. Furthermore, screening for GSW is typically not part of ambulatory care patient history, although there have been recent implementations of firearm screening in some mental health clinics.^7^ In addition to ICD codes, patients’ GSW experience may be captured in unstructured acEHR data, such as providers’ clinical notes. However, identification of patients with GSW by clinical notes may be challenging even with recent advances in artificial intelligence and natural language processing and may be unfeasible for many healthcare systems owing to limited time and financial resources.

Thus, continuity of patient care as well as the study of short- and long-term physical and behavioral health outcomes from GSWs may then be limited by the paucity of diagnostic codes for GSW in acEHR. To estimate the extent of this limitation, the primary objective of this study was to determine the percentage of patients with a GSW diagnosis (ICD code) in their acEHR from among all patients with an Oregon Medicaid claim with an acute-care GSW diagnosis and an observed follow-up ambulatory-care visit. The secondary objective was to explore patient demographic and clinical factors associated with a patient having a GSW diagnosis in their acEHR. These findings will help determine what data are available and what needs to be addressed to improve the study of health sequelae of nonfatal GSWs in the U.S.

Past studies on mental health outcomes after GSWs have been conducted with data from regional healthcare systems,^4,8,9^ public or employer-based insurance claims,^5,10,11^ police reports,^8^ or surveys.^12^ Because non-fatal GSWs are predominantly from assault occurring in low-income and minoritized populations, it is important that study data reflect these low-resource populations who often have limited access to ambulatory care services.^13^ Likewise, with the adoption and expansion of the Affordable Care Act, an estimated 20% of the U.S. population aged <65 years became Medicaid enrollees.^14^ Medicaid was the payer of over 50% of the patients hospitalized for GSWs in Greater New Orleans in 2016 through 2019.^15^ Therefore, those covered by Medicaid represent an important segment of the U.S. population who receive treatment for the sequelae of GSW-related injuries in the safety-net outpatient setting. The data used in this study consist of outpatient primary and behavioral healthcare electronic health record (EHR) from a nationwide network of safety-net clinics and Oregon Medicaid claims data, representing a population more at risk for having a GSW.

## METHODS

### Study Population

Ambulatory care patient EHR data were provided by OCHIN, Inc., a nonprofit consultancy dedicated to advancing health and well-being for everyone. OCHIN’s ADVANCE Data Warehouse is the nation’s largest and most comprehensive database on community-based health care, housing acEHR for a multistate network of over 2,000 member clinics across 40 states.^16^ OCHIN member health clinics provide primary and behavioral health care to patients in underserved communities regardless of their ability to pay. Sixty-four percent of patients in the ADVANCE Data Warehouse are uninsured, publicly insured, or underinsured.

Data were first limited to all member clinics associated with patients with an ICD for GSWs from either encounter diagnoses or the patient’s problem list. ICD codes for GSWs (which do not include injuries due to paintballs, BBs, rubber bullets, or pellets) are given in Appendix Table 1 (available online).^17^ Then, all records for all patients with at least 1 encounter at 1 or more of those member clinics anytime in the study time period (January 1, 2012–December 31, 2022) were included in the acEHR data (*n*=5,095,378 patients). A deidentified data set consisting of demographics, encounter characteristics, and ICD diagnosis codes (encounter based and medical history/problem list) for these patients was provided.

Acute GSW data were obtained from Oregon Medicaid claims data, with permission of the Oregon Health Authority, for those OCHIN patients described earlier who also had an Oregon Medicaid claim with a GSW diagnosis code during the study period (*n*=1,004). A deidentified data set consisting of IP and outpatient Oregon Medicaid claims data for these patients was provided. In general, linking data across health systems or insurance providers is a difficult task. In this study, OCHIN provided a unique patient identifier that enabled linkage of Oregon Medicaid data to OCHIN’s acEHR.

There were 1,004 patients with Oregon Medicaid claim for an acute GSW and at least 1 observed ambulatory encounter. From these, patients were excluded if (1) the Medicaid claim place of service was other than that of an ED, IP hospital, urgent care, or ambulance or (2) they died within 2 calendar years of acute GSW or (3) they had no ambulatory encounter after the acute care GSWs. This study only included Medicaid claims with a place of service in a hospital setting to determine how robust ICD codes are at identifying patients with GSW in subsequent care; it is assumed that if care of acute GSW event occurred in an ambulatory setting, a GSW ICD code would be present in the acEHR. If a patient had multiple acute-care GSW records occurring in more than 1 calendar year (*n*=22), the first year of occurrence was used as the index year. Because the deidentified set contained only the year of the acute GSW index event and EHR encounter, the time sequence of acute and ambulatory events that occurred in the same year could not be determined when acEHR data did not have a GSW diagnosis; these patients were removed from the data set (Figure 1).

### Measures

Patient demographics (sex, age, race and ethnicity) and characteristics (Charlson score, insurance status, income, homeless status, and injury severity of GSW) are given for all patients. Race and ethnicity were combined and categorized as Hispanic, any race, or unknown race (referred to as Hispanic in the remaining parts of this paper); non-Hispanic Black, non-Hispanic White, or non-Hispanic other when ethnicity was non-Hispanic or unknown; and unknown when both race and ethnicity were unknown. The Charlson score is an OCHIN-specific variant of the well-known Charlson Comorbidity Index, which is a weighted measure of chronic comorbid disease burden.^18^ The proprietary version of the Charlson Comorbidity Index includes added weights for transplantation history, inflammatory bowel disease, seizures, sickle cell anemia, hemophilia, muscular dystrophy, Down syndrome, cystic fibrosis, Tày-Sachs disease, developmental delay, mental retardation, cerebral palsy, autism, schizophrenia, bipolar disorder, and drug or alcohol abuse. Used by permission (Charlson M, personal communication, 2017). Insurance type, income, and homeless status were collected at each ambulatory care encounter. If a patient’s status was unknown at every recorded encounter, the value unknown was assigned to that measure. Otherwise, insurance status (insured, uninsured) and income (above poverty level, poverty level or below) were aggregated into 1 value by taking the mode of the known values. Homeless status (yes, no) was aggregated by defining a patient as homeless if they were ever recorded as homeless during the study period, else not. Injury severity of GSW was measured by the Trauma Mortality Prediction Model (TMPM), a continuous variable computed using recorded anatomic injuries from all Medicaid GSW claims.^19,20^ The TMPM uses regression modeling to estimate empiric probabilities of death from each of the injuries in the ICD, Ninth Revision with Clinical Modification and ICD-10-CM lexicons. Patients without an injury severity measure (those without trauma codes in their Medicaid claims, *n*=29) were assigned the minimum value in the data set (9.57 × 10^5^).

The primary outcome of this study was the percentage of patients with acute-care GSW with an acEHR GSW diagnosis. The secondary outcome was to determine patient characteristics associated with a GSW diagnosis in acEHR, through both bivariate tests and logistic regression.

### Statistical Analysis

Bivariate statistics (chi-square for categorical variables or Wilcoxon rank sum for non-normally distributed continuous variables) were used to assess differences between groups. As such, the chi-square was used to test for differences between groups of patients with and without a GSW code in their acEHR for the categorical variables of sex, race and ethnicity, income, and homeless status, whereas the rank sum test was used for the continuous variables of age at GSW, Charlson score, the number of calendar years between acute GSW index year and year of first observed follow-up, and injury severity (given by the TMPM). The threshold for statistical significance was *p*<0.05, and all analyses were 2 sided.

A multivariable logistic regression model was used to estimate the strength of association between covariates and having GSW diagnosis in acEHR. The modeling process began with all covariates: age, sex, race and ethnicity, income, homeless status, Charlson score, insurance status, number of calendar years between acute GSW index year and year of first observed follow-up, and the logit-transformed TMPM measure of injury severity. The method of purposeful selection was then used to determine inclusion of predictors, interactions among predictors, and nonlinear relationships between continuous predictors and the outcome defined by splines and fractional polynomials.^21^ Final model specification was based on the model’s ability to discriminate patients with an ambulatory ICD code for GSW (evaluated by the area under the receiver operating characteristic [ROC] curve) and the model fit (evaluated using the Hosmer–Lemeshow goodness of fit).^21,22^ The most parsimonious model was presented. All data manipulation, statistical testing, and logistic regression modeling was performed using Stata (Version 18) and Python (Version 3.10.14).^23,24^ This study received IRB review and approval from Norwich University.

## RESULTS

From the 1,004 patients with Oregon Medicaid claim for an acute GSW and at least 1 observed ambulatory encounter, (1) *n*=320 were excluded because the Medicaid claim place of service was other than that of an ED, IP hospital, urgent care, or ambulance; (2) *n*=8 were excluded because they died within 2 calendar years of acute GSW; and (3) *n*=218 were excluded because they had no ambulatory encounter after the acute-care GSW. Patients without an acEHR GSW diagnosis whose time sequence of events could not be determined because acute and ambulatory events occurred in the same year (*n*=61) were also removed from the data set. The final study population included 397 patients. Of these 397 patients with an acute-care claim for GSW and a subsequent observed ambulatory encounter, 34.3% (*n*=136) had a GSW diagnosis in their acEHR. As seen in Table 1, those with an ICD for GSW in their acEHR were slightly younger and had a higher percentage of female patients, although neither of these differences were statistically significant. The group with GSW ICD codes tended to be seen within the same year as their acute GSW, with more severe injuries (as indicated by a higher TMPM value) than those without an ICD for GSW in their acEHR. There were more patients whose homeless status is unknown in the group without a GSW diagnosis in their acEHR than those with GSW diagnosis. There were no differences in the Charlson score, income, and insurance status between the 2 groups. Race and ethnicity, number of calendar years between acute GSW and follow-up care, and injury severity as measured by the TMPM were univariately associated with GSW diagnosis in acEHR, each being statistically significant with *p*<0.05.

Table 2 shows the results of the multivariable logistic regression modeling after using the method of purposeful selection. Homeless status and income were not included in the model because their inclusion did not improve model performance or fit, and there were no clinically relevant reasons to retain them. For the same reasons, interactions and nonlinear relationships between continuous predictors and the outcome were not included in the model. Thus, the resultant model controlled for age, sex, race and ethnicity, Charlson score, insurance status, number of calendar years to follow-up care, and injury severity. The model showed good discrimination (area under the ROC curve=0.79) and calibration (Hosmer–Lemeshow goodness of fit=14.31, df=15, *p*=0.35). A plot of the ROC curve for the logistic regression model is given in Figure 2, together with a diagonal line representing the ROC curve of a random classifier.

The logistic regression model showed that each additional calendar year between acute-care GSW and ambulatory encounter decreased the odds of having an acEHR GSW diagnosis by 75%. The model also showed that higher injury severity (TMPM) was associated with increased odds of having an acEHR GSW diagnosis. Both Black, non-Hispanic and Hispanic patients were more likely to have an acEHR GSW diagnosis than White, non-Hispanic patients.

## DISCUSSION

Patient health data across health care settings are required for both providing whole-person care on an individual basis and evaluating the full scope of firearm injury on health across the U.S. population. In this study, most patients with an acute GSW did not have GSW indicated in their acEHR during a subsequent ambulatory encounter—neither in their encounter diagnosis nor in their medical history/problem list. There are numerous possible explanations for this finding, including that ICD codes for GSW are largely limited to acute bodily trauma and, therefore, often not applicable to ambulatory settings. Moreover, assessing and documenting GSW are typically not part of the standard of care in ambulatory settings. As a result, ambulatory providers could miss important information related to patient health, and research on the full trajectory of health after GSW will remain limited.

Potential strategies to identify patients with GSW in acEHR include linkage of records across healthcare settings, although this is difficult given the U.S. multipayer system. Identifying history elements such as GSW, which are often not included in typical clinical screening questionnaires, would require screening by providers whose time with patients is already severely limited. In addition, automated identification by clinical notes may not capture GSW events if the patient did not share or the provider did not ask.

### Limitations

Methodologic limitations include the low percentage of patients with acute GSW who received care at an OCHIN clinic, which resulted in a small study sample and inability to control for differences between clinics; the lack of granular time data to more accurately describe follow-up ambulatory care; and the inclusion of only 1 state in the analysis. Furthermore, the ICD codes used to identify GSWs are from the External Causes of Injury and Poisoning code group and are, therefore, mechanism of injury codes; such codes may not be required for reimbursement.

## CONCLUSIONS

In one state for which linkage of acute care GSW events from Medicaid data with ambulatory health center data was possible, it was found that most patients with an acute-care GSW event did not have a GSW diagnosis in their acEHR. Findings from this novel study suggest that ambulatory care providers often do not know of their patient’s GSW history. Strategies to document prior GSW in ambulatory EHRs should be developed to support whole-person care and to support the study of health outcomes, healthcare utilization, and healthcare costs associated with GSW.

## Supplementary Material

AJPM_Cartwright_Supplemental

Supplemental materials associated with this article can be found in the online version at https://doi.org/10.1016/j.amepre.2025.108254.

## Figures and Tables

**Figure 1.**
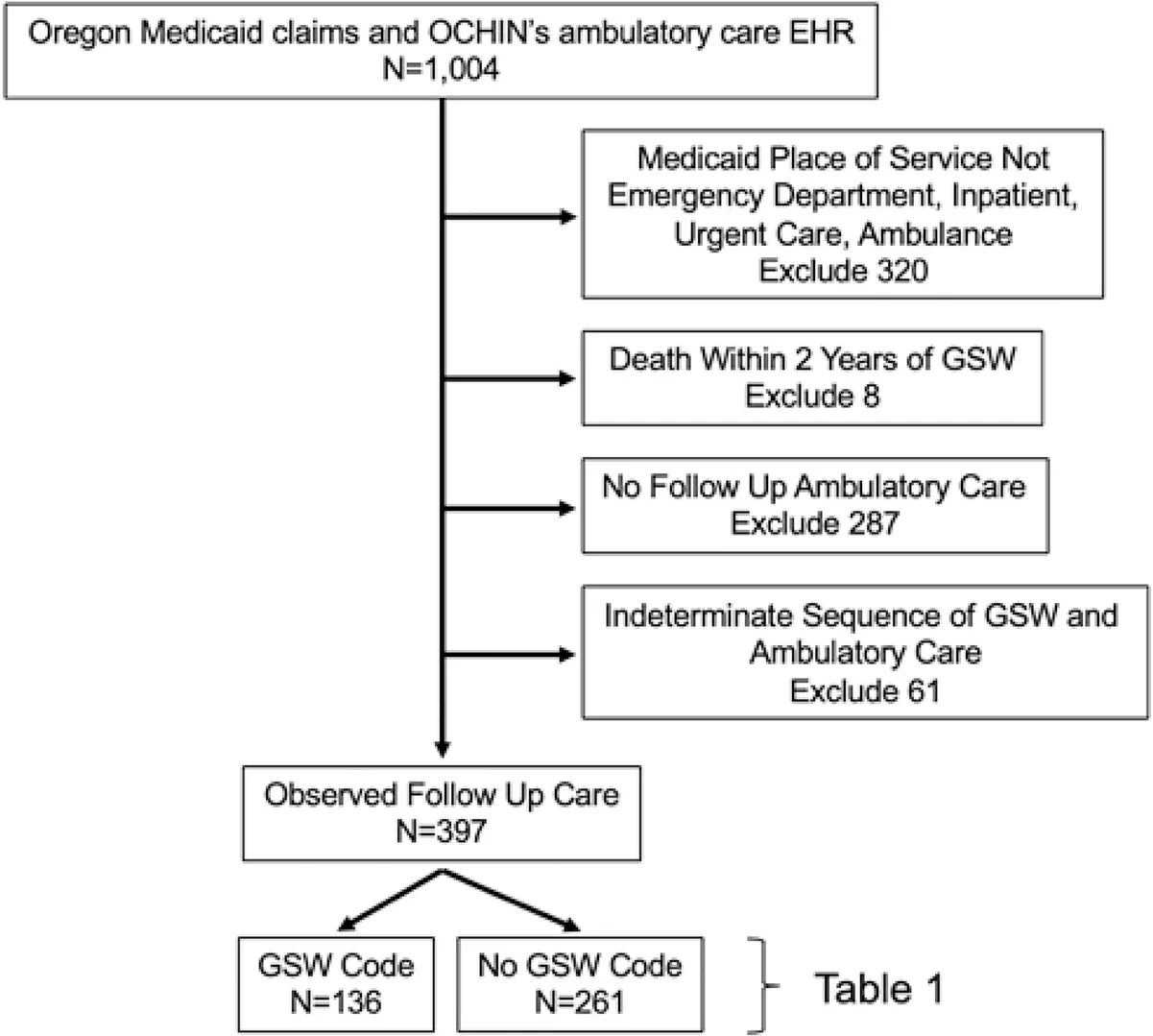
Flow diagram.

**Figure 2.**
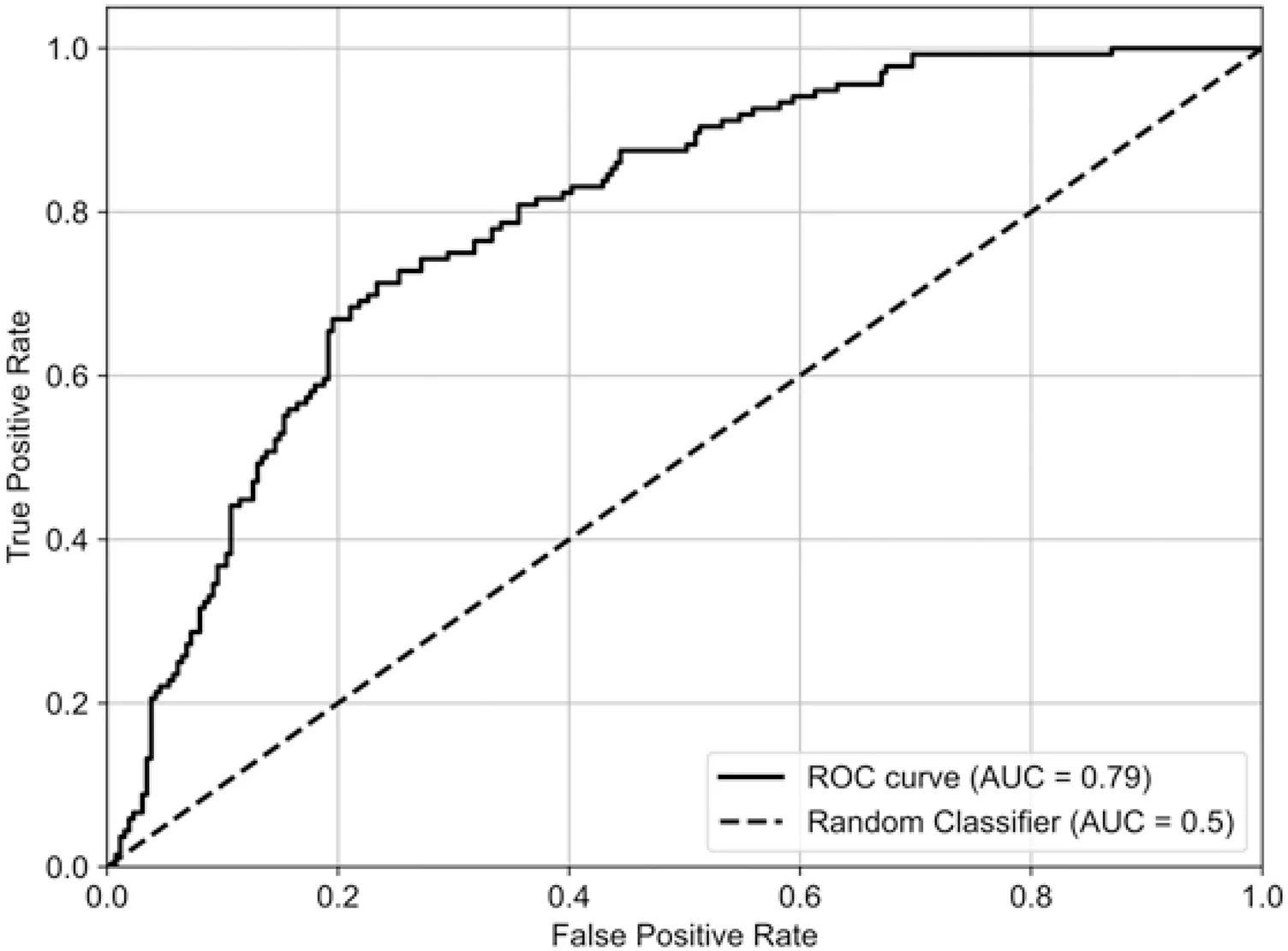
ROC curve for the multivariable logistic regression model. ROC, receiver operating characteristic.

**Table 1.** Characteristics of Patients With Acute-Care GSW With Observed Follow-Up in Ambulatory Care With and Without ICD Code for GSW in Their acEHR

Characteristic	GSW code in acEHR	Without GSW code in acEHR	*p*-value

*n* (%)	136 (34.3)	261 (65.7)	
Age at GSW, years			0.09
Minimum	1	2	
Median (IQR)	29.5 (21–38.25)	31 (24–42)	
Maximum	66	74	
Sex, *n* (%)			0.13
Female	27 (19.9)	35 (13.4)	
Male	109 (80.1)	226 (86.6)	
Race and ethnicity, *n* (%)			**0.001**
White, non-Hispanic or ethnicity unknown	61 (44.9)	151 (57.9)	
Black/African American, non-Hispanic or ethnicity unknown	39 (28.7)	39 (14.9)	
Hispanic, any race or race unknown	24 (17.6)	30 (11.5)	
Other	*	29 (11.1)	
Unknown	*	12 (4.6)	
Income, *n* (%)			0.86
Poverty level or below	104 (76.5)	193 (73.9)	
Above poverty level	17 (12.5)	36 (13.8)	
Unknown	15 (11.0)	32 (12.2)	
Homeless, *n* (%)			0.05
Yes	26 (19.1)	53 (20.3)	
No	104 (76.5)	178 (68.2)	
Unknown	6 (4.4)	30 (11.5)	
Charlson score			0.53
Minimum	0	0	
Median (IQR)	3 (1–6)	3 (0–5)	
Maximum	16	15	
Insurance status, *n* (%)			>0.99
Insured	123 (90.4)	237 (90.8)	
Uninsured	13 (9.6)	24 (9.2)	
Number of calendar years between acute GSW and observed follow-up	0	0	**<0.001**
Minimum	0	0	
Median (IQR)	[0,0]	[0,2]	
Maximum	3	9	
Injury severity (TMPM)			**0.006**
Minimum	0.0004	0.0001	
Median (IQR)	0.021 (0.010–0.042)	0.013 (0.008–0.028)	
Maximum	0.374	0.799	
N/A	4	25	

*Note*: Boldface indicates statistical significance (*p*<0.05).

Asterisks are used to suppress cell counts <11.

acEHR, ambulatory-care electronic health record; GSW, gunshot wound; N/A, not available; TMPM, Trauma Mortality Prediction Model.

**Table 2.** Multivariable Logistic Regression Model of GSW ICD Code in OCHIN acEHR

Variable	Odds Ratio	95% CI	P-value
Age at GSW	0.99	0.97 – 1.01	0.33
Sex			
Female	reference	reference	
Male	0.71	0.38 – 1.33	0.29
Race and Ethnicity			
White, non-Hispanic	reference	reference	
Black, non-Hispanic	2.32	1.26 – 4.28	**0.01**
Hispanic	2.08	1.01 – 4.26	0.05
Other	0.91	0.39 – 2.12	0.82
Unknown	0.32	0.06 – 1.64	0.17
Charlson Score	1.02	0.94 – 1.11	0.63
Insurance Status			
Insured	reference	reference	
Uninsured	0.80	0.36 – 1.77	0.58
Number of calendar years between acute GSW and observed follow-up	0.25	0.15 – 0.43	**< 0.001**
Injury Severity (TMPM)	1.23	1.07 – 1.42	**< 0.05**

*Note*: Boldface indicates statistical significance (*p*<0.05).

acEHR, ambulatory-care electronic health record; ICD, International Classification of Diseases; GSW, gunshot wound.
