# Supplementary material for: Continuity of Firearm Injury Documentation From Acute Care to Ambulatory Care Among Medicaid Enrollees in Oregon
Source: Am J Prev Med. Author manuscript; Available in PMC 2026 Sep 30. (PMC13624957; doi:10.1016/j.amepre.2025.108254)
Supplement: Supplementary file 1 [file NIHMS2180360-supplement-AJPM_Cartwright_Supplemental.pdf]

Continuity of firearm injury documentation from acute care to ambulatory care among Medicaid enrollees in Oregon

Natalie Cartwright, PhD
Alan Cook, MD, MS, FACS
Frances Biel, MPH, MS
Kerime Toksu, MA
David Hosmer, PhD
Turner Osler, MD
Megan Hoopes, MPH
Nicole Cook PhD, MPA

American Journal of Preventative Medicine

Appendix Table 1. International Classification of Disease (ICD) codes used to identify firearm injury. The given ICD-09-CM codes are complete matches while those for ICD-10-CM are any codes that start with the given letters and numbers, e.g. W32.0XXA falls into W32*. These codes do not include injuries due to paintballs, BBs, rubber bullets or pellets.

	Firearm Injury Codes
ICD-09-CM	E922.0, E922.1, E922.2, E922.3, E922.8, E922.9, E955.0, E955.1, E955.2, E955.3, E955.4, E955.9, E965.0, E965.1, E965.2, E965.3, E965.4, E970, E979.4, E985.0, E985.1, E985.2, E985.3, E985.4, E991.2, E991.9
ICD-10-CM	W32*, W33*, W34.00*, W34.09*, W34.10*, W34.19*, X72*, X73*, X74.8*, X74.9*, X93*, X94*, X95.8*, X95.9*, Y22*, Y23*, Y24.8*, Y24.9*, Y35.00*, Y35.01*, Y35.02*, Y35.09*, Y36.43*, Y38.4*
